# Effect of increased protein intake and exogenous ketosis on body composition, energy expenditure and exercise capacity during a hypocaloric diet in recreational female athletes

**DOI:** 10.3389/fphys.2022.1063956

**Published:** 2023-01-13

**Authors:** Charlotte Hiroux, Moniek Schouten, Isabelle de Glisezinski, Chantal Simon, François Crampes, Peter Hespel, Katrien Koppo

**Affiliations:** ^1^ Department of Movement Sciences, Exercise Physiology Research Group, KU Leuven, Leuven, Belgium; ^2^ INSERM, UMR 1048, Institute of Metabolic and Cardiovascular Diseases, Obesity research Laboratory, Paul Sabatier University, Toulouse, France; ^3^ Physiological Functional Exploration Department, Toulouse University Hospitals, Toulouse, France; ^4^ Carmen INSERM U1060, Human Nutrition Research Centre of Rhône-Alpes, NRA U1235, University of Lyon, Lyon, France

**Keywords:** ketones, resting metabolic rate, appetite, performance, VO_2_max

## Abstract

**Introduction:** Since low body weight is an important determinant of success in many sports such as gymnastics, martial arts and figure skating, athletes can benefit from effective weight loss strategies that preserve muscle mass and athletic performance. The present study investigates the effects of increased protein intake and exogenous ketosis on body composition, energy expenditure, exercise capacity, and perceptions of appetite and well-being during a hypocaloric diet in females.

**Methods:** Thirty-two female recreational athletes (age: 22.2 ± .5 years; body weight: 58.3 ± .8 kg; BMI: 20.8 ± .2 kg·m^−2^) underwent 4 weeks of 30% caloric restriction and were randomized to receive either an increased daily amount of dietary protein (PROT, ∼2.0–2.2 g protein·kg^−1^·day^−1^), 3 × 20 g·day^−1^ of a ketone ester (KE), or an isocaloric placebo (PLA). Body composition was measured by DXA, resting energy expenditure (REE) by indirect calorimetry, exercise capacity during a VO_2_max test, appetite hormones were measured in serum, and perceptions of general well-being were evaluated *via* questionnaires.

**Results:** The hypocaloric diet reduced body weight by 3.8 ± .3 kg in PLA, 3.2 ± .3 kg in KE and 2.4 ± .2 kg in PROT (P_time_<.0001). The drop in fat mass was similar between treatments (average: 2.6 ± .1 kg, P_time_<.0001), while muscle mass was only reduced in PLA and KE (average: .8 ± .2 kg, P_time_<.05), and remained preserved in PROT (P_interaction_<.01). REE [adjusted for lean mass] was reduced after caloric restriction in PLA (pre: 32.7 ± .5, post: 28.5 ± .6 kcal·day^−1^·kg^−1^) and PROT (pre: 32.9 ± 1.0, post: 28.4 ± 1.0 kcal·day^−1^·kg^−1^), but not in KE (pre: 31.8 ± .9, post: 30.4 ± .8 kcal·day^−1^·kg^−1^) (P_interaction_<.005). Furthermore, time to exhaustion during the VO_2_max test decreased in PLA (by 2.5 ± .7%, *p* < .05) but not in KE and PROT (P_interaction_<.05). Lastly, the perception of overall stress increased in PLA and PROT (*p* < .05), but not in KE (P_interaction_<.05).

**Conclusion:** Increased protein intake effectively prevented muscle wasting and maintained exercise capacity during a period of caloric restriction in female recreational athletes. Furthermore, exogenous ketosis did not affect body composition, but showed its potential in weight management by preserving a drop in exercise capacity and REE and by improving overall stress parameters during a period of caloric restriction.

## Introduction

Since low body weight is an important determinant of success in many sports, athletes try to reduce body weight either to comply with the physical appearance standards, to compete in a lower weight class or to increase physical performance. In order to reduce body weight a negative energy balance is required. This can be achieved either by cutting energy intake, by increasing energy expenditure, or a combination of both. Since athletes are subjected to high training loads with a concomitant high energy expenditure, the obvious way to lower body weight is *via* energy intake restriction. However, sustained hypocaloric diets might come with certain pitfalls as in general they induce not only losses in fat mass but also in lean mass in a ratio of approximately 3:1 (Weinheimer et al., 2010). A small fraction of the reduction in lean mass is accounted for by the drop of bone mass, but the majority results from muscle wasting. It is well established that caloric restriction impairs muscle protein synthesis (Pasiakos et al., 2010; 2013; Areta et al., 2014) and, on the other hand, enhances protein breakdown (Carbone et al., 2013; 2014). This negative balance results in muscle wasting which is detrimental to exercise performance, and often also elevates injury risk (Fogelholm, 1994). A strategy to circumvent this problem is to increase the daily intake of high-quality protein (for a review on dietary protein, see (Phillips and Van Loon, 2011)). Indeed, a high-protein diet in young, physically active volunteers effectively prevented muscle atrophy during short-term caloric restriction (Mettler et al., 2010; Pasiakos et al., 2013; Longland et al., 2016). These observations underpin current recommendations with regard to protein intake during weight loss in athletic populations (Helms et al., 2014; Manore, 2015; Hector and Phillips, 2018). Nonetheless, it is important to note that these recommendations largely result from observations in young males, while well-controlled weight loss studies in females are underrepresented. It was shown in overweight and obese females that hypocaloric high-protein diets were successful in reducing fat mass while simultaneously preserving lean mass (Piatti et al., 1994; Josse et al., 2011; Campbell and Meckling, 2012). However, whether the same effect occurs in already lean female athletes remains to be determined. Some prospective case studies showed promising experiences of female figure competitors who were able to preserve muscle mass during a caloric deficit by increasing protein intake (Halliday et al., 2016; Petrizzo et al., 2017; Rohrig et al., 2017; Tinsley et al., 2019). However, these athletes also adhered to a strenuous resistance training regimen combined with several performance enhancing supplements, making it difficult to define the effects of increased protein intake *per se*. Furthermore, prospective case studies do not allow to determine any causal relationships, and therefore high quality intervention trials in female athletes are required.

Ketone bodies (i.e. β-hydroxybutyrate, (βHB), acetoacetate (AcAc) and acetone) may provide an alternative strategy to counteract muscle wasting during caloric restriction. Ketone bodies are lipid-derived compounds which are produced in the liver in response to low blood glucose and insulin levels (Evans et al., 2017). They were shown to exert anti-catabolic actions under stress conditions. More specifically, ketone salt infusion decreased urinary nitrogen excretion during prolonged starvation in obese individuals (Sherwin et al., 1975; Pawan and Semple, 1983) and reduced net muscle protein loss during lipopolysaccharide-induced inflammation in healthy volunteers (Thomsen et al., 2018). Also the anabolic potential of ketone bodies has been demonstrated, as ketone salt infusion suppressed leucine oxidation and stimulated muscle protein synthesis in healthy volunteers (Nair et al., 1988). Additionally, oral ingestion of the ketone ester (R)-3-hydroxybutyl (R)-3-hydroxybutyrate post-exercise in young healthy volunteers enhanced stimulation of the mTORC1 axis as shown by increased phosphorylation of S6K1 and 4E-BP1 (Vandoorne et al., 2017). Overall, the anticatabolic and anabolic potential of ketone bodies might make them as effective as an increased protein intake to counteract muscle loss during a period of caloric restriction.

To this background, we performed a double-blind, placebo-controlled study to compare the effects of exogenous ketosis with those of an increased daily protein intake (i.e. the prevailing strategy to obtain weight loss in athletic populations) on body composition and exercise capacity during rapid weight loss. We hypothesized that exogenous ketosis can facilitate maintenance of muscle mass, as well as promote exercise capacity during rapid weight loss in already lean female recreational athletes. We selected this specific study population to better validate the existing dietary recommendations in female recreational athletes.

## Methods

### Subjects

Thirty-three healthy, young female recreational athletes were recruited according to the following inclusion criteria: between 18 and 35 years old; exercise participation for at least 6 h per week; body fat percentage between 16% and 25% (based on 12 skinfolds, see below); stable body weight for at least 3 months prior to the start of the study; consistent use of oral contraceptives; non-smoking. Candidate subjects were excluded for participation if they had an obsessive pursuit of thinness confirmed by the Eating Disorder Inventory 3 (Clausen et al., 2011). Health status was evaluated by a medical questionnaire and physical examination prior to enrollment in the study. One subject dropped out for reasons that were unrelated to the study protocol. Baseline characteristics of the subjects who completed the full study protocol (*n* = 32) were: age: 22.2 ± .5 years; body weight: 58.3 ± .8 kg; height: 1.67 ± .01 m; BMI: 20.8 ± .2 kg·m^−2^; body fat percentage: 21.4 ± .6%, and were similar between the experimental groups. The study was approved by the KU Leuven Biomedical Ethics Committee (S61133). Subjects gave written consent to participate after being fully informed of all procedures and potential risks associated with the study.

### Study design and experimental conditions

**FIGURE 1:**
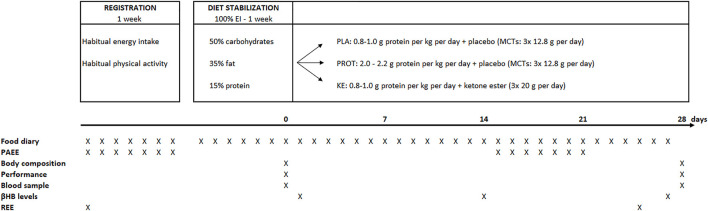
Study design and timing of measurements. *EI, e*nergy intake; MCTs, medium chain triglycerides; PAEE, physical activity-related energy expenditure; βHB, β-hydroxybutyrate; REE, resting energy expenditure.

A schematic overview of the double-blinded placebo-controlled study design is presented in Figure 1. Subjects were instructed to maintain their habitual level of physical activity, as well as their exercise training routine throughout the full study period. The protocol started with a baseline registration week during which the subjects recorded their habitual diet and physical activities. They completed an on-line food diary (Mijn Eetmeter, Stichting Voedingscentrum Nederland, https://mijn.voedingscentrum.nl). To improve the accuracy of the dietary analysis, the subjects received a kitchen scale to weigh all consumed foods and energy-containing drinks. Daily physical activity level was monitored using an accelerometer (Actigraph, wGT3X, Pensacola, United States) together with a training diary (see below). After the baseline registration week, the subjects were enrolled in a diet stabilization week. They received a fully-standardized diet containing 50% carbohydrates, 35% fat and 15% protein to deliver 100% of their estimated ‘optimal energy intake’. Optimal energy intake was determined as the mean of daily energy intake and energy expenditure. Daily energy intake was taken from the 7-day food diary filled out during the registration week. Daily energy expenditure was calculated as the sum of resting energy expenditure, which was measured by indirect calorimetry (see below), physical activity-induced energy expenditure, which was obtained from the accelerometer data during the registration week, and estimated diet-induced thermogenesis. Following the stabilization period, a 4-week caloric restriction period was started. Energy intake was reduced to 70% of the optimal energy intake in the stabilization week, including the 291 kcal∙day^−1^ delivered by the supplements. Subjects with similar body weight, % body fat and energy intake were first allocated to triplets, whereafter they were randomly split over the three experimental conditions: i) increased daily protein intake with placebo supplementation (PROT: *n* = 10), ii) ketone ester supplementation (KE: n = 11) and iii) placebo supplementation (PLA: *n* = 11). Subjects in PLA and KE received .8–1.0 g protein∙kg^−1^∙day^−1^ while subjects in PROT received 2.0–2.2 g protein∙kg^−1^∙day^−1^. Protein intake was distributed over the different meals and snacks during the day. Fractional energy intake *via* carbohydrates was 50% in all groups. Energy intake *via* fat was adjusted according to protein intake in order to obtain 30% caloric restriction in all subjects. Three times daily, i.e. immediately before breakfast, lunch, and dinner, the subjects ingested a ketone ester or an isocaloric placebo (see below). Supplement intake was blinded to both the subjects and the investigators. At the start (pretest) and at the end (posttest) of the caloric restriction period, the subjects participated in an experimental session which involved body composition and resting energy expenditure (REE) measurements, blood sampling, exercise testing, and questionnaires addressing general well-being.

### Nutritional protocol

During the stabilization and the caloric restriction period, the subjects received an individual food plan. All meals, snacks and drinks were provided by the investigators. The subjects were instructed not to consume any foods or drinks other than prescribed by the study protocol, except water or other zero-calorie drinks. To avoid vitamin or mineral deficiencies, the subjects received a daily supplement at breakfast (Omnibionta three Defense, Omnibionta, Overijse, Belgium). Subjects also completed a food diary to register any deviation from the nutritional plan. Table 1 shows the energy and macronutrient intake of the subjects during the study period, taking into account reported non-compliances. Protein intake on average was .97 ± .01 g·kg^−1^·day^−1^ in PLA and KE, vs. 2.10 ± .01 g·kg^−1^∙day^−1^ in PROT.

**TABLE 1:** Macro-nutrient composition of the experimental diets. Data are mean ± SEM. Energy intake refers to the energy intake exclusive 291 kcal∙day^−1^ delivered by the ketone and placebo supplements. Macronutrient composition is expressed in g per day. In week 0, the subjects received 100% of the estimated optimal energy intake in the form of a well-balanced mixed diet. In weeks one to four, total energy intake, i.e. nutrition plus supplement drinks, was reduced by 30% (.8–1.0 g protein ∙ kg BW^−1^∙day^−1^) and subjects received either placebo (PLA: *n* = 11), an increased amount of dietary protein (PROT: *n* = 10) or a ketone ester (KE: *n* = 11). 1 kcal = 4.18 kJ.

PLA		PROT	KE
Energy intake (kcal∙day^−1^)			
Week 0	2,328 ± 49	2,446 ± 59	2,354 ± 65
Week 1	1,361 ± 29	1,451 ± 34	1,394 ± 49
Week 2	1,354 ± 36	1,447 ± 30	1,390 ± 47
Week 3	1,372 ± 33	1,437 ± 38	1,384 ± 51
Week 4	1,358 ± 34	1,412 ± 37	1,346 ± 42
Protein (g∙day^−1^)			
Week 0	85 ± 2	90 ± 2	86 ± 3
Week 1	56 ± 2	119 ± 2	56 ± 2
Week 2	56 ± 2	120 ± 2	56 ± 2
Week 3	54 ± 1	118 ± 2	55 ± 2
Week 4	56 ± 1	116 ± 2	55 ± 1
Carbohydrates (g∙day^−1^)			
Week 0	307 ± 7	322 ± 8	313 ± 9
Week 1	169 ± 4	180 ± 5	172 ± 5
Week 2	169 ± 5	180 ± 5	172 ± 6
Week 3	170 ± 6	178 ± 6	170 ± 6
Week 4	172 ± 5	180 ± 7	171 ± 6
Fat (g∙day^−1^)			
Week 0	92 ± 2	96 ± 3	92 ± 2
Week 1	51 ± 1	28 ± 2	53 ± 3
Week 2	51 ± 1	28 ± 2	53 ± 2
Week 3	53 ± 2	28 ± 2	54 ± 3
Week 4	50 ± 2	25 ± 2	49 ± 2

#### Ketone ester and placebo supplements

Subjects in KE received a 20 g ketone ester (R)-3-hydroxybutyl (R)-3hydroxybutyrate (TdeltaS Ltd, Thame, Oxfordshire, UK) three times daily. We used this orally absorbable ketone ester because it was proven to be safe and well-tolerated in humans (Clarke et al., 2012) and shown to be more effective than ketone salts to raise blood βHB and with less incidence of gastrointestinal problems (Stubbs et al., 2017). Supplements were taken immediately before breakfast, lunch and dinner. Subjects in PLA and PROT received an isocaloric placebo drink containing 12.8 g pure medium chain triglycerides (Now Foods, Bloomingdale, United States). To match the taste and appearance of the placebo drink with the ketone ester, bitter sucrose octaacetate (Sigma-Aldrich, Bornem, Belgium) was added. Supplement drinks were blinded for both subjects and researchers.

#### Experimental session

During the stabilization period, the subjects participated in a familiarization session in order to habituate to the exercise testing procedures (see below) and thereby reduce potential learning effects between the pretest and the posttest. Subjects were instructed to refrain from any strenuous physical activity for at least 48 h prior to each experimental session. On the evening before the pre- and posttest, the subjects consumed a standardized light meal (∼430 kcal, 61% carbohydrates, 11% fat, 28% protein) between 7 and 10 p.m., whereafter only water was allowed till the next morning. They arrived fasted at the laboratory between 7 and 11 a.m. Upon arrival, after a toilet visit, body weight was measured (Henk Maas, PUE C/31, Veen, The Netherlands) and body composition was assessed by a whole-body dual-energy X-ray absorptiometry (DXA) scan (Discovery W, Hologic Inc, Bedford, MA). Subcutaneous fat mass was also assessed *via* 12 skinfold measurements (biceps, triceps, subscapular, supra-iliac, midaxillary, iliac-crest, abdomen, chin, anterior thigh, posterior thigh, lateral calf and medial calf) using a Harpenden skinfold caliper (Baty International Ltd, West Sussex, UK). Subsequently, a fasting blood sample was taken from a cubital vein (Venoject, Tokyo, Japan) and plasma or serum were separated by centrifugation and stored at -20°C until analyzed. Subjects completed a number of questionnaires addressing general mood status, perception of satiety, and gastro-intestinal discomfort (see below). The subjects then received a standardized light breakfast (∼268 kcal, 54% carbohydrates, 5% fat, 41% protein). Ninety min after breakfast, the exercise testing was started. Subjects first performed a series of 3 strength and power tests. Handgrip strength was measured with the dominant hand using a handgrip dynamometer (Jamar, J00105, Lafayette, United States). Due to a hand injury, one subject in PROT was excluded from this analysis. Explosive strength was evaluated by countermovement jumps (CMJ) on a force platform (SMARTJUMP, Fusion sport, Nottingham, UK). Maximal isometric force of the knee extensors was measured in the dominant leg at a knee angle of 135° on an isokinetic dynamometer (Hespel et al., 2001). For each test, five attempts were allowed with 1 min rest and the mean of the three best performances was used for further analyses. Finally, a maximal incremental VO_2_max test on a cycle ergometer was performed (Avantronic Cyclus II, Leipzig, Germany). Initial workload was set at 50 W for 5 min and was increased by another 20 W per min until volitional exhaustion. Respiratory gas exchange was measured continuously (Cortex MetaLyzer II, Leipzig, Germany) and the highest oxygen uptake measured over a 30 s period was noted as the maximal oxygen uptake rate (VO_2_max). Two minutes after exhaustion, a blood sample (5–10 µL) was taken from an earlobe for lactate determination (Lactate Pro2, Arkray, Japan). Because of technical issues VO_2_max data from one subject in PLA is absent. Four weeks later, the subjects returned to the laboratory for the posttest, which was identical to the pretest. For each subject, the experimental diet and supplementation was maintained till the day before the posttest, and tests were done on the same day of the week and the same time of the day as for the pretests.

#### Resting and physical activity-related energy expenditure

REE and substrate oxidation were measured during the registration period (baseline) and at the end of the caloric restriction period (day 24 or 25, posttest). Subjects were instructed to refrain from any intense physical exercise from 24 h prior to the measurement. The evening before, they received a standardized light meal (∼430 kcal, 61% carbohydrates, 11% fat, 28% protein) before 8 p.m. whereafter they fasted till next morning, yet water was allowed at libitum. Subjects were instructed not to perform any physical activity on the morning of the REE registration. They arrived in the laboratory between 7 and 9 a.m., with identical timing for the pretest and the posttest. After a toilet visit, 24 h urine collection was started. Subjects then rested on a bed in a dark and quiet room for 1 h. Subsequently, REE and resting carbohydrate and lipid oxidation were measured during two 20 min episodes with a 10 min break in between using a calibrated gas analyzer with a canopy hood (Quark RMR Cosmed, Rome, Italy). Following each 20 min measurement, a post-calorimetric simulation test was performed to correct for potential drifts that emerged in the measured VO_2_ and VCO_2_. The simulation consisted of sending a well-defined gas flow (coming from the same gas bottle as was used for the initial calibration with 16% O_2_ and 5% CO_2_) *via* a mass flow meter to the gas analyzer. Three simulations were performed in order to obtain three levels of FeCO_2_ (.7; .85; 1). Each simulation lasted 2 min, resulting in a total duration of 6 min for the post-calorimetric test. During the simulation, the same ventilation was used as that during the REE measurement. Finally, measured VO_2_ and VCO_2_ values were corrected according to the regression line that was established between the measured values and the theoretical values of the simulation. Two subjects were excluded from these analyses because they exhibited abnormally high REE values due to fever (KE: n = 1; PROT: n = 1). Physical activity-related energy expenditure (PAEE) was determined using an accelerometer (Actigraph, wGT3X, Pensacola, United States) in conjunction with a training diary. Each exercise training activity was registered in a training diary including type of exercise performed, exercise duration, and rate of perceived exertion (RPE) according to a 15-point Borg scale (Borg, 1990). Furthermore, the subjects were equipped with the accelerometer during the full registration week (pretest measurement) and during week three of the caloric restriction period (posttest measurement). The third week, instead of the fourth week of the caloric restriction was chosen because of the exercise limitations imposed in the direct approach of the posttest and REE measurement in week 4. Subjects were instructed to wear the accelerometer on the right hip during days and nights and whenever it was removed, the reason and time window had to be noted in the training diary. Only days including more than 10 h of daytime registration were included in the analyses. PAEE was estimated based on an activity-specific model coupled to an automatic activity/posture recognition algorithm as previously described (Bastian et al., 2015; Garnotel et al., 2018). When subjects removed the accelerometer during training, PAEE was calculated from the exercise duration, the MET-value of the sports activity, and the RPE. The PAEE of non-registered sports activities was added to the daily PAEE estimated by the accelerometer. Subjects who were unable to perform their habitual training routine for >3 days due to disease were excluded from this analysis (KE: *n* = 2; PROT: *n* = 1). Total Energy Expenditure (TEE) was calculated as the sum of REE, PAEE and 10% diet induced thermogenesis using the following formula: $TEE=\left(REE+PAEE\right)/0.9$ .

#### Questionnaires addressing satiety, well-being and gastro-intestinal comfort

Perception of satiety was evaluated in the fasted state using a 0–10 Likert visual analogue scale (VAS) (Woods et al., 2018). Satiety (maximal score 20) was scored as the sum of scores on the questions ‘how full do you feel’ and ‘how satisfied do you feel’. Gastro-intestinal discomfort was evaluated in the fasted state using a 0–8 Likert scale questionnaire addressing upper and lower abdominal problems, and systemic problems (Pfeiffer et al., 2009). Total gastro-intestinal discomfort (maximal score 96) was scored as the sum of scores on the ‘upper abdominal problems’ (heartburn, bloating, nausea and vomiting, maximal score of 32), ‘lower abdominal problems’ (intestinal cramps, abdominal pain, flatulence, diarrhea, maximal score of 32) and ‘systemic problems’ (dizziness, headache, muscle cramps, urge to urinate, maximal score of 32). General mood status was assessed by the Recovery Stress Questionnaire for Athletes (RESTQ-36) (Kallus and Kellmann, 2001). An ‘overall stress score’ (maximal score 54) was calculated as the sum of scores for ‘general stress’, ‘social stress’ and ‘fatigue’ subscales. An ‘overall recovery’ score (maximal score 54) was calculated as the sum of scores on the ‘social recovery’, ‘general well-being’ and ‘sleep quality’ subscales.

#### Blood and urine analyses

Commercially available ELISA kits were used to determine total ghrelin (EZGRT-89K, Merck, Darmstadt, Germany), leptin (RD191001100, Biovendor, Brno, Czech Republic) and GDF15 (DGD150, R&D, Minneapolis, United States) in serum. Serum free triiodothyronine (T3), free thyroxine (T4) and thyroid-stimulating hormone (TSH) were measured *via* electrochemiluminescence using cobas e 801 (Roche Diagnostics, Mannheim, Germany). Furthermore, on days 1, 14 and 27 of the caloric restriction period, capillary blood samples for βHB determination (GlucoMen Lx plus meter with Lx β-ketone sensor strips, Menarini Diagnostics, Firenze, Italy) were taken from an earlobe immediately before and 1 h after each supplement intake before meals. From the 24 h urine samples collected in the context of the REE determination, total volume was registered and aliquots were stored at -20°C until analyzed. Total nitrogen concentration was assayed according to the Dumas method using a continuous-flow elemental analyzer isotope ratio mass spectrometer (ANCA-2020, Europa Scientific, Crewe, UK) as previously described (De Preter et al., 2007). Total urinary nitrogen output was used to determine 24 h protein oxidation rate.

#### Statistical analyses

Statistical analyses were performed using GraphPad Prism version 8.0.0 for Windows (GraphPad Software, San Diego, California United States). Differences between the experimental groups at baseline were analyzed using a one way repeated measures analysis of variance (ANOVA). Differences between the experimental groups over time were analyzed using a two way repeated measures ANOVA. In case of a significant group × time interaction, Bonferroni *post hoc* tests were performed to further specify the differences. Statistical significance was set as *p* < .05. Reported *p*-values that refer to observed main effects are specified as P_time,_ P_group_ and P_interaction_, other *p*-values refer to the *post hoc* analyses. All results are expressed as mean ± SEM. Effect sizes were reported as eta squared (η^2^). Sample size was determined based on earlier studies investigating the effect of a high-protein hypocaloric diet on muscle wasting (primary endpoint of this study) in normal-weight subjects (Mettler et al., 2010; Pasiakos et al., 2013).

## Results

**FIGURE 2:**
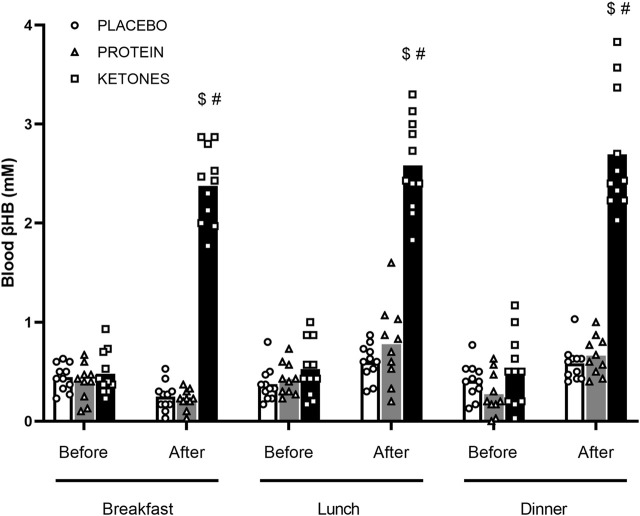
Diurnal blood β-hydroxybutyrate. Subjects were involved in a 4-week hypocaloric diet (30% energy restriction, .8–1.0 g protein ∙ kg BW^−1^∙day^−1^) and received either placebo (PLA: *n* = 11), an increased amount of dietary protein (PROT: n = 10) or a ketone ester (KE: *n* = 11). Data are represented as means (white bars: PLA; grey bars: PROT; black bars: KE) and individual values (circles: PLA; triangles: PROT; squares: KE). Supplements were ingested daily immediately before breakfast (8 am), lunch (12 pm) and dinner (6 pm). βHB (mM) was measured immediately before and 1h after supplement intake on day 1, 14 and 27 during the supplementation period. Because diurnal βHB levels were similar between day 1, 14 and 27, only mean values of the 3 days are reported. In case of significant interaction, *post hoc* differences are shown as $: significantly different from the corresponding value before breakfast (p < .05), #: significantly different from PLA and PROT (p < .05).

*Diurnal blood βHB levels* (Figure 2)—Fasted blood βHB levels were <.5 mM in each group. In PLA and PROT, diurnal βHB concentrations did not significantly change compared with the fasted levels before breakfast. However in KE, supplement intake before meals increased blood βHB on average to ∼2.5 mM (range: 1.8–3.8 mM) by 1 h after intake (*p* < .0001). βHB levels returned to fasted baseline levels before the next supplement intake 4–6 h later. In all groups, diurnal βHB levels were similar between day 1, 14 and 27 of the supplementation period.

*Body composition* (Table 2)—For all body composition measurements, pretest values were similar between the experimental groups. Compared with the pretest, body weight declined after 4 weeks of caloric restriction in all groups (P_time_<.0001). Body weight reductions were similar in PLA and KE with an average loss of 3.8 kg (range: −6.0 to −2.5 kg) and 3.2 kg (range: −4.6 to −.7 kg), respectively, while body weight reductions in PROT (−2.4 kg, range: −3.1 to −1.3 kg) were smaller compared to PLA (P_interaction_<.005). Irrespective of the experimental condition, a large part of this body weight decrement was due to a decrease in fat mass (on average: −2.6 kg, range: −4.0 to −.9 kg, P_time_<.0001). The drop in fat mass also translated into a ∼16% smaller sum of skinfolds in all groups (on average: pre: 141 ± 4 mm, post: 119 ± 4 mm, P_time_<.0001). The caloric restriction period on average reduced lean mass by .9 ± .3 kg in PLA (*p* < .005) and .7 ± .2 kg in KE (*p* < .05), but not in PROT (+.3 ± .3 kg, P_interaction_<.01). Compared with the pretest, bone mineral content and density declined by ∼2% in all groups (P_time_<.0001).

**TABLE 2:** Effect of increased protein intake and exogenous ketosis on body composition during a hypocaloric diet. Data are mean ± SEM. Subjects were involved in a 4-week hypocaloric diet (30% energy restriction, .8–1.0 g protein ∙ kg BW^−1^∙day^−1^) and received either placebo (PLA: *n* = 11), an increased amount of dietary protein (PROT: *n* = 10) or a ketone ester (KE: *n* = 11). Body composition was measured using DXA before (pretest) and at the end (posttest) of the caloric restriction period. Significant main effects are shown in bold. In case of significant interaction, *post hoc* differences are shown as $: significantly different from the corresponding value at pretest (*p* < .05). Effect sizes are reported as eta squared (η^2^).

PLA		PROT	KE	P (Group); η^2^	P (Time); η^2^	P (Group x Time); η^2^
Body weight (kg)						
Pretest	58.7 ± 1.4	57.8 ± 1.5	58.4 ± 1.4	.990; .07	**<.0001**; .93	**.0042**; .31
Posttest	55.0 ± 1.3 $	55.4 ± 1.5 $	55.2 ± 1.3 $			
Fat mass (kg)						
Pretest	12.6 ± .6	12.4 ± .7	12.4 ± .7	.995; .01	**<.00001**; .94	.397; .06
Posttest	9.7 ± .6	9.8 ± .6	10.0 ± .6			
Lean mass (kg)						
Pretest	46.2 ± 1.4	45.4 ± 1.1	46.0 ± 1.2	.992; .05	**.004**; .25	**.008**; .28
Posttest	45.2 ± 1.2 $	45.6 ± 1.1	45.2 ± 1.2 $			
Bone mineral content (g)						
Pretest	2,220 ± 83	2,232 ± 76	2,370 ± 97	.398; .88	**<.0001**; .50	.714; .02
Posttest	2,178 ± 74	2,187 ± 75	2,312 ± 85			
Bone mineral density (g∙cm^−2^)						
Pretest	1.14 ± .02	1.16 ± .02	1.18 ± .03	.377; .77	**<.0001**; .42	.873; .01
Posttest	1.12 ± .02	1.14 ± .02	1.17 ± .02			

**FIGURE 3:**
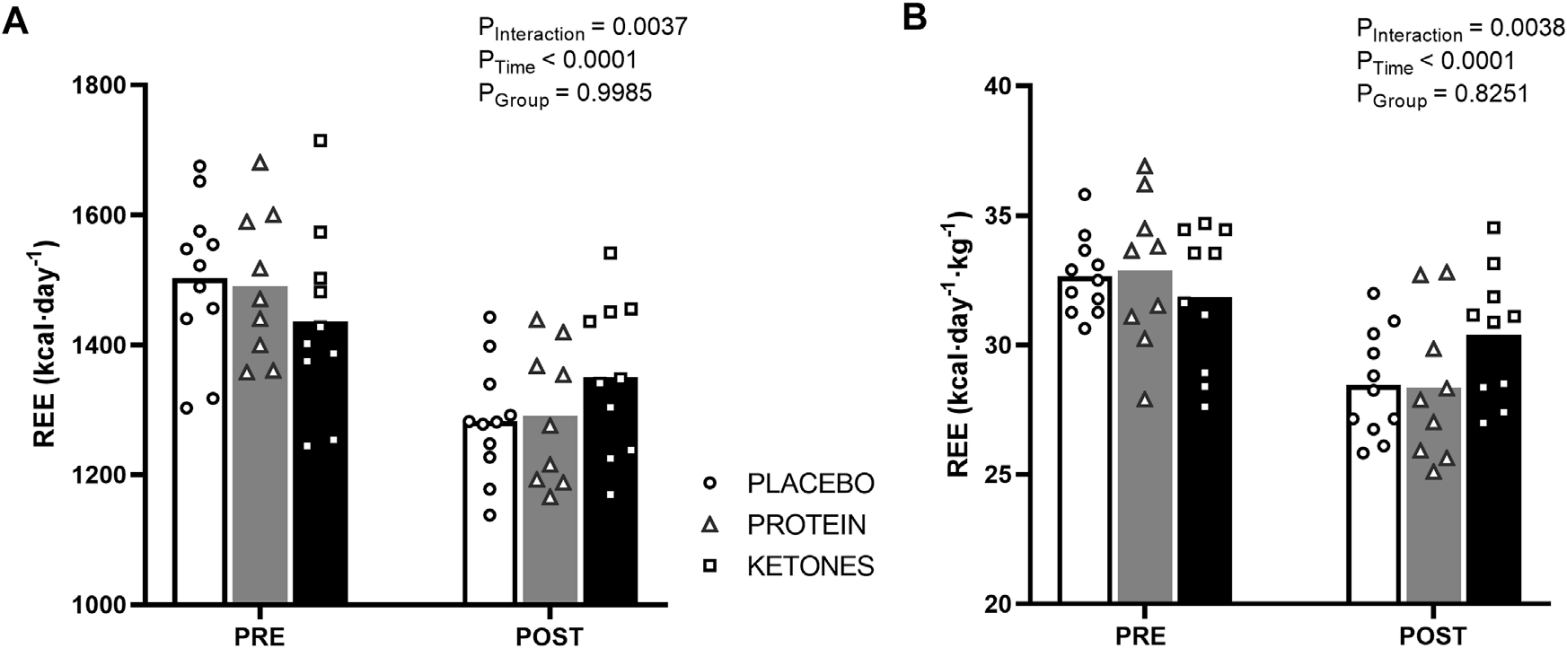
Effect of increased protein intake and exogenous ketosis on resting energy expenditure during a hypocaloric diet. Subjects were involved in a 4-week hypocaloric diet (30% energy restriction, .8–1.0 g protein ∙ kg BW^−1^∙day^−1^) and received either placebo (PLA: *n* = 11), an increased amount of dietary protein (PROT: *n* = 9) or a ketone ester (KE: *n* = 10). Data are represented as means (white bars: PLA; grey bars: PROT; black bars: KE) and individual values (circles: PLA; triangles: PROT; squares: KE). Absolute resting energy expenditure **(A)** was measured by indirect calorimetry in the fasted state before (pretest) and at the end (posttest) of the caloric restriction period. The hypocaloric diet reduced absolute REE in all groups, but to a lesser extend in KE. When REE values were adjusted for lean mass **(B)**, REE was only reduced in PLA and PROT, and was preserved in KE.

*Energy expenditure* (Figure 3; Table 3)—In the pretest, REE on average was ∼1,500 kcal∙day^−1^ and was similar between the experimental conditions. The hypocaloric diet reduced REE in all groups (P_time_<.0001). Declines in REE were similar in PLA and PROT with an average of -221 ± 23 kcal and -200 ± 39 kcal, respectively, while reductions in KE were smaller with an average of -85 ± 22 kcal; P_interaction_<.005). When absolute REE values were adjusted for lean mass, REE was only reduced in PLA and PROT (*p* < .001), but was preserved in KE (P_interaction_<.005). Absolute PAEE was on average 822 ± 32 kcal∙day^−1^ in the pretest and declined to 706 ± 31 kcal∙day^−1^ at the end of the caloric restriction period (P_time_<.001), but was not different between the groups at any time. Consequently, also absolute TEE declined at the end of the caloric restriction period from 2,556 ± 43 to 2,229 ± 45 kcal∙day^−1^ (P_time_<.0001), without differences between groups at any time. PAEE and TEE values adjusted for lean mass also showed a similar reduction over time in all groups (data not shown). Fasting carbohydrate oxidation rates were not affected by the caloric restriction period, whereas fasting lipid oxidation rates decreased on average by ∼16% compared with the pretest (P_time_<.005) in all groups. Protein oxidation rates were similar between the groups in the pretest and markedly increased during the caloric restriction period in PROT (∼41%, *p* < .001), whereas it decreased in KE (∼34%, *p* < .005) and did not significantly change in PLA (P_interaction_<.0001).

**TABLE 3:** Effect of increased protein intake and exogenous ketosis on physical activity-related energy expenditure, total energy expenditure and substrate oxidation at rest during a hypocaloric diet. Data are mean ± SEM. Subjects were involved in a 4-week hypocaloric diet (30% energy restriction, .8–1.0 g protein ∙ kg BW^−1^∙day^−1^) and received either placebo (PLA), an increased amount of dietary protein (PROT) or a ketone ester (KE). Physical activity-related energy expenditure (PAEE; PLA: n = 11; PROT: *n* = 9; KE: *n* = 9) was measured during the registration period and during week three of the caloric restriction period. Total energy expenditure (TEE; PLA: *n* = 11; PROT: *n* = 9; KE: *n* = 9) was calculated as the sum of REE, PAEE and 10% diet induced thermogenesis using the following formula: TEE=(REE + PAEE)/0.9. Substrate oxidation (PLA: *n* = 11; PROT: n = 9; KE: *n* = 10) was measured by indirect calorimetry in the fasted state before (pretest) and at the end (posttest) of the 4-week caloric restriction period. Significant main effects are shown in bold. In case of significant interaction, *post hoc* differences are shown as $: significantly different from the corresponding value at pretest (*p* < .05), #: significantly different from PLA (*p* < .05), *: significantly different from KE (*p* < .05). Effect sizes are reported as eta squared (η^2^).

PLA		PROT	KE	P (Group); η^2^	P (Time); η^2^	P (Group x Time); η^2^
PAEE (kcal∙day^−1^)						
Pretest	826 ± 45	792 ± 67	847 ± 64	.547; .14	**.0006**; .37	.601; .04
Posttest	677 ± 49	679 ± 32	769 ± 59			
TEE (kcal∙day^−1^)						
Pretest	2,588 ± 45	2,537 ± 63	2,538 ± 114	.694; .09	**<.0001**; .70	.100; .16
Posttest	2,177 ± 57	2,178 ± 44	2,342 ± 115			
Carbohydrate oxidation (mg∙min^−1^)						
Pretest	94 ± 8	80 ± 8	78 ± 6	.184; .14	.265; .05	.473; .05
Posttest	84 ± 7	62 ± 11	81 ± 13			
Fat oxidation (mg∙min^−1^)						
Pretest	54 ± 4	57 ± 3	56 ± 5	.414; .12	**.002**; .30	.120; .15
Posttest	45 ± 2	41 ± 3	54 ± 5			
Protein oxidation (mg∙min^−1^)						
Pretest	44 ± 3	47 ± 4	43 ± 3	**<.0001**; .69	.749; .00	**<.0001**; .59
Posttest	37 ± 2	67 ± 4 $,#,*	28 ± 3 $			

*Exercise performance* (Table 4)—VO_2_max in the pretest was 2.73 ± .13, 2.48 ± .07 and 2.68 ± .07 L·min^−1^ in PLA, PROT and KE, respectively (*p* > .05). The hypocaloric diet similarly decreased absolute VO_2_max by ∼4–9% in each group (P_time_<.0001). However, because body weight declined during caloric restriction, VO_2_max relative to body weight did not significantly change. Time to exhaustion in the VO_2_max test decreased by 2.5 ± .7% from the pretest to the posttest in PLA (*p* < .05), but was unaffected in KE and PROT (P_interaction_<.05). Peak heart rate dropped by ∼5–7 bpm (*p* < .001) in PLA and PROT, but not in KE (P_interaction_<.0001). Peak blood lactate levels on average were also slightly lower in the posttest than in the pretest in all groups (P_time_<.05). In the pretest, handgrip strength was 34 ± 1 kg (range: 24–47 kg, *n* = 31), countermovement jump height was 26 ± 1 cm (range: 15–39 cm, *n* = 32), and maximal isometric knee extension torque was 162 ± 5 Nm (range: 99–212 Nm, *n* = 32). Values were unchanged following the caloric restriction period and were similar between groups at any time (see Supplementary Table S1).

**TABLE 4:** Effect of increased protein intake and exogenous ketosis on performance in the VO_2_max test during a hypocaloric diet. Data are mean ± SEM. Subjects were involved in a 4-week hypocaloric diet (30% energy restriction, .8–1.0 g protein ∙ kg BW^−1^ ∙ day^−1^) combined with placebo (PLA: n = 11), an increased amount of dietary protein (PROT: n = 10) or a ketone ester (KE: n = 11). An incremental VO_2_max test was performed before (pretest) and at the end (posttest) of the caloric restriction period. Significant main effects are shown in bold. In case of significant interaction, *post hoc* differences are shown as $: significantly different from the corresponding value at pretest (*p* < .05), *: significantly different from KE (*p* < .05). Effect sizes are reported as eta squared (η^2^).

PLA		PROT	KE	P (Group); η^2^	P (Time); η^2^	P (Group x Time); η^2^
VO_2_max (l∙min^−1^)						
Pretest	2.73 ± .13	2.48 ± .07	2.68 ± .07	.181; .51	**<.0001**; .46	.153; .13
Posttest	2.47 ± .10	2.35 ± .08	2.57 ± .07			
VO_2_max (ml∙min^−1^∙kg^−1^)						
Pretest	46.0 ± 1.8	43.2 ± 1.5	46.0 ± 1.4	.271; .07	.144; .47	.404; .06
Posttest	44.3 ± 1.6	42.1 ± 1.6	46.2 ± 1.7			
Time to exhaustion (min)						
Pretest	14.3 ± .3	13.3 ± .3	14.2 ± .4	.080; .80	.449; .02	**.020**; .24
Posttest	13.9 ± .3 $	13.3 ± .3	14.3 ± .3			
Maximal heart rate (bpm)						
Pretest	188 ± 3	186 ± 3	188 ± 2	.107; .67	**<.0001**; .44	**<.0001**; .48
Posttest	182 ± 2 $,∗	179 ± 2 $,∗	190 ± 2			
Lactate after 2 min (mmolˑl^−1^)						
Pretest	5.6 ± .5	6.7 ± .8	6.7 ± .7	.178; .44	**.013**; .20	.397; .06
Posttest	4.7 ± .4	5.6 ± .7	6.5 ± .6			

*Hormonal parameters* (Table 5)—Irrespective of the experimental condition, serum leptin and free T3 decreased during the caloric restriction period (P_time_<.01), whereas TSH, serum ghrelin, GDF15 and free T4 remained stable throughout the study period in all treatment groups.

**TABLE 5:** Effect of increased protein intake and exogenous ketosis on hormonal parameters during a hypocaloric diet. Data are mean ± SEM. Subjects were involved in a 4-week hypocaloric diet (30% energy restriction, .8–1.0 g protein ∙ kg BW^−1^∙day^−1^) and received either placebo (PLA: *n* = 11), an increased amount of dietary protein (PROT: *n* = 9) or a ketone ester (KE: *n* = 11). Hormonal parameters were measured before (pretest) and at the end (posttest) of the caloric restriction period. Significant main effects are shown in bold and effect sizes are reported as eta squared (η^2^).

PLA		PROT	KE	P (Group); η^2^	P (Time); η^2^	P (Group x Time); η^2^
Leptin (ng∙ml^−1^)						
Pretest	4.1 ± .8	4.4 ± .8	3.2 ± .6	.417; .18	**<.0001**; .62	.386; .06
Posttest	1.4 ± .2	2.2 ± .6	1.5 ± .4			
Ghrelin (pg∙ml^−1^)						
Pretest	686 ± 88	783 ± 113	854 ± 89	.878; .02	.140; .07	.577; .04
Posttest	676 ± 123	642 ± 135	645 ± 152			
GDF15 (pg∙ml^−1^)						
Pretest	378 ± 43	428 ± 74	548 ± 54	**.043**; .26	.940; .00	.864; .01
Posttest	402 ± 35	422 ± 86	520 ± 31			
Free T3 (pmol∙l^−1^)						
Pretest	4.4 ± .2	4.5 ± .2	4.7 ± .2	.134; .31	**.002**; .28	.246; .09
Posttest	3.6 ± .3	4.0 ± .4	4.5 ± .3			
Free T4 (pmol∙l^−1^)						
Pretest	14.4 ± .5	14.7 ± .5	15.8 ± .7	**.040**; .33	.653; .01	.527; .04
Posttest	13.6 ± .7	14.9 ± .9	15.9 ± .4			
TSH (mU∙l^−1^)						
Pretest	2.6 ± .3	2.4 ± .3	2.8 ± .3	.893; .06	.078; .10	.857; .01
Posttest	2.9 ± .6	2.8 ± .5	3.0 ± .3			

*Questionnaires addressing satiety, stress and recovery status, and gastro-intestinal comfort*–The hypocaloric diet increased the scoring of ‘overall stress’ in PLA and PROT (on average: pre: 9.9 ± .7, post: 15.5 ± 1.2, *p* < .05), but not in KE (pre: 10.9 ± 1.4, post: 11.6 ± 2.2, P_interaction_<.05). Scoring of injury was unaffected by the hypocaloric diet in PLA and KE (on average: pre: 5.0 ± 10.5, post: 5.8 ± .5), but increased in PROT (pre: 4.6 ± .5, post: 8.1 ± 1.0, *p* < .01, P_interaction_<.05). The hypocaloric diet decreased perception of ‘overall recovery’ (on average: pre: 38.4 ± 1.2, post: 32.8 ± 1.6, P_time_<.005) and ‘being in shape’ (on average: pre: 13.0 ± .4, post: 10.2 ± .6, P_time_<.0001) similarly in all groups. Subjective feelings of ‘satiety’ showed a significant time effect (P_time_<.05) with a tendency for a time × group interaction (P_interaction_ = .097), where exogenous ketosis increased subjective feelings of satiety (*p* < .05) during the hypocaloric diet. Although not significant, the same trend was observed for PROT but not for PLA. Systemic and lower gastro-intestinal discomfort was almost absent in the pretest (on average: 1.9 ± .5 and 1.8 ± .7, respectively) and did not change during the caloric restriction period in all groups. However, upper gastro-intestinal discomfort slightly increased from the pretest to the posttest in PROT (pre: .2 ± .1, post: 2.0 ± .8, *p* < .05), but not in PLA and KE (on average: pre: .9 ± .4, post: .5 ± .3, P_interaction_<.05).

## Discussion

Low body weight is an important determinant of success in many sports. However, sustained hypocaloric diets generally induce undesired losses in lean mass. As such, effective dietary strategies that preserve muscle mass and athletic performance are essential. Therefore, the current study aimed to investigate the effect of high protein intake as well as to evaluate the effectiveness of exogenous ketosis to preserve muscle mass during caloric restriction in female recreational athletes. Young lean females were enrolled in a fully-controlled weight loss program either combined with an increased intake of dietary protein, ketone ester supplementation or placebo.

Results of the current study indicate that increased protein intake fully inhibited muscle wasting in lean females during a period of caloric restriction, as was previously shown in males (Mettler et al., 2010; Pasiakos et al., 2013). Increased protein intake also preserved exercise capacity as time to exhaustion during an incremental VO_2_max test remained unchanged following the caloric restriction period. On the other hand, increased protein intake was unable to prevent a decrease in REE and PAEE and did not affect appetite or stress regulation. Although exogenous ketosis did not inhibit muscle wasting, it preserved exercise capacity as effectively as increased protein intake. Ketone ester supplementation also preserved a drop in REE and in overall stress parameters, but did not affect appetite regulation.

### Body composition

Because current recommendations with regard to weight loss in the context of athletic performance are largely based on studies in young fit males, we conducted the current intervention study in young lean females. Although we did not directly compare the effects of caloric restriction between males and females, we performed a study with a protocol which was very similar to an earlier study in young males (Mettler et al., 2010). Overall, body composition changes produced by the hypocaloric diet - in combination with increased protein intake or not - were equivalent to the findings reported by Mettler et al. (Mettler et al., 2010) and others (Pasiakos et al., 2013). This corroborates earlier findings showing that muscle protein turnover, in the basal state as well as in response to protein feeding, is similar between young females and males (Fujita et al., 2007; Smith et al., 2009). However, in the context of elite sports, especially in sports where aesthetics and the degree of leanness are important determinants of performance, females often enroll in even more extreme weight loss regimens than males, causing potential hormonal dysregulations that might result in bone demineralization (Joy et al., 2014). In the current study, the 4-week caloric restriction period on average reduced bone mineral content and density by ∼2%, which is in line with earlier reports (Soltani et al., 2016). It has been proposed that an increased rate of protein intake may help to preserve bone mass during caloric restriction (Tang et al., 2014; Wright et al., 2019), though this was not confirmed in the current study. Interestingly, our research group previously reported that consistent ketone ester supplementation during a period of endurance training overload slightly increased bone mineral content in young male volunteers (Poffé et al., 2019). However, the increase in bone mineral content was accompanied by an increased caloric intake in the KE group, resulting in a caloric balance compared to a caloric deficit in the control group. As such, the beneficial effect of exogenous ketosis on bone metabolism might have been driven by changes in caloric intake. Indeed, in the current study where all groups were subjected to a 30% caloric deficit, exogenous ketosis could not counter a drop in bone mineral content. These observations underpin the link between energy balance and bone metabolism which was previously reported by others (Confavreux et al., 2009; Lombardi et al., 2012; DeLoughery and Dow, 2020).

### Energy expenditure

REE typically declines during a period of caloric restriction, due to a drop in body weight. Such a drop may eventually impair additional body weight loss or impede maintenance of low body weight (Pasman et al., 1999; Rochon et al., 2011; Varkevisser et al., 2019). Since REE is highly impacted by lean body mass (Ravussin et al., 1986) it is often suggested that preserving lean body mass during caloric restriction is a successful strategy to counteract the drop in REE. However, several studies that combined caloric restriction with resistance training to preserve lean body mass still reported declines in REE (Schwartz and Doucet, 2010; Hunter et al., 2015; Stratton et al., 2020). Accordingly, increased daily protein intake in the conditions of the current study was unable to inhibit the decline in REE despite preserving muscle mass. As such, factors other than lean body mass may determine REE during caloric restriction (Schwartz and Doucet, 2010). This is supported by the finding in the present study that exogenous ketosis, which did not prevent muscle wasting, was able to preserve REE. That exogenous ketosis might affect REE has been proposed in a previous study from our lab in which participants supplemented with ketones during an overtraining period maintained an energetic balance as opposed to an energetic deficiency in the placebo group without differences in body weight between the groups (Poffé et al., 2019). Here it was speculated that a decrease in REE in the control group explained the absence of a drop in body weight.

### Exercise performance

The 4-week caloric restriction period impaired performance during an incremental VO_2_max test. Time to exhaustion was reduced which was associated with lower peak heart rates and blood lactate. Nonetheless, the reduction in time to exhaustion was blunted in PROT, likely due to the preservation of muscle mass. Surprisingly, also in KE time to exhaustion was unaffected by the caloric restriction period. The preserved exercise capacity induced by exogenous ketosis is in line with a previous study from our lab where chronic ketone supplementation during a 3-week overload training protocol improved exercise tolerance during the last training week (Poffé et al., 2019). It should be noted that in this study exogenous ketosis also increased voluntary energy intake, predominantly from carbohydrates, questioning its potential direct effect on exercise tolerance. In contrast, in the current study, participants were restricted to a prescribed diet omitting any variation in spontaneous eating behavior or voluntary energy intake. As such, the effects in the current study are attributed to a direct effect of ketone ester supplementation. Importantly, participants performed the VO_2_max tests in the absence of ketosis, which excludes potential acute effects of ketones on exercise performance. Nevertheless, chronic ketone ester supplementation might have induced adaptations in muscle tissue such as increased angiogenesis (Weis et al., 2022) and thereby improved time to exhaustion compared to PLA. Unfortunately, this hypothesis goes beyond the scope of the current study and future studies are required to clarify the mechanism(s) behind the improved exercise capacity induced by ketones.

### Satiety, stress and recovery status and gastro-intestinal comfort

Long-term success in weight management largely depends on psychological factors, such as the perception of hunger and satiety (Hansen et al., 2019). Therefore, we looked at the effect of protein and ketone ester intake on appetite hormone regulation and perception for a given degree of caloric restriction. The prevailing opinion that high-rate protein intake increases satiety to a greater extent than carbohydrate and fat consumption (Paddon-Jones et al., 2008), was not confirmed by our findings as PROT had no impact on satiety hormones or perceived appetite. The effects of ketone ingestion on appetite and satiety are not fully elucidated yet. Previous studies show that acute ketone intake may suppress hunger and desire to eat (Stubbs et al., 2018; Poffé et al., 2020; 2021; Poffe et al., 2021) *via* various mechanisms, including central actions in the brain (Laeger et al., 2012), reductions in circulating ghrelin (Stubbs et al., 2018; Poffé et al., 2020) and GDF15 (Poffe et al., 2021) levels and ketoacidosis (Poffé et al., 2021). In contrast, we previously demonstrated that post-exercise ketone ester administration during short-term endurance training overload not only impacted appetite hormone regulation, but also altered spontaneous eating behavior by stimulating voluntary energy intake (Poffé et al., 2019). However, the results of the present study show that exogenous ketosis altered neither circulating ghrelin, leptin or GDF15 levels, nor affected satiety perception. The current study design obviously excludes changes in voluntary eating behavior as well as any acute effect of exogenous ketosis on perceived appetite and appetite hormones since blood samples and questionnaires were taken early morning in the fasted state, 8–10 h after the last ketone ester dose, i.e. at equally low circulating ketone levels (<.5 mM) in all groups.

Besides perception of hunger and satiety, psychological factors such as overall well-being and stress play a crucial role in weight loss maintenance. In fact it was shown that comfort eating, induced by negative emotions and stress, is a common cause for weight regain, especially in females (Zellner et al., 2006; Sainsbury et al., 2019). Whereas overall stress increased during the caloric restriction period in PLA and PROT, it remained stable in KE. Our group previously showed that exogenous ketosis was able to counteract the sympathetic overdrive induced by an intense training period by inhibiting the increase in catecholamine excretion (Poffé et al., 2019). These findings suggest a mechanism *via* which ketone ingestion may have reduced overall stress in the current study, thereby proposing a potential role for exogenous ketosis to facilitate low body weight maintenance.

## Limitations and future directions

The subjects who enrolled in the current study were young lean females who participated in daily physical activity and training. Their body fat percentage was low, i.e. ∼21.4%, but still significantly higher than in most elite female athletes (≤15%). It is well-known that further energy restriction in very lean females submitted to strenuous athletic training often results in physiological dysregulations which are referred to as the ‘relative energy deficiency in sport’ (RED-S) (Manore et al., 2007; Mountjoy et al., 2014). Based on the current results, we cannot exclude that under such extreme catabolic conditions (Manore et al., 2007; Loucks et al., 2011) exogenous ketosis may have an impact on body composition.

Hydration status was not determined, which may be considered to be a limitation of the current study, since DXA results are influenced by total body water content. However, considering the high level of control in this study (i.e. fully provided diet and strict control of physical activity), it can be argued whether potential small differences in water retention would be significant enough to influence the interpretation of the DXA results.

The exercise tests included in the current protocol are based on well-controlled laboratory settings. Therefore, generalization to functional performance during specific sports, such as gymnastics, martial arts or figure skating, remains presumptuous. Nevertheless, the finding that increased protein intake effectively blunts muscle wasting during a period of caloric restriction in young lean females adds valuable insights to the field of female exercise physiology.

## Conclusion

This is the first study to investigate the effect of increased protein intake and exogenous ketosis on body composition, energy expenditure and exercise capacity during a hypocaloric diet in young lean recreational female athletes. Our observations demonstrate that also young lean females benefit from increased protein intake to effectively prevent muscle wasting and maintain exercise capacity during a period of caloric restriction. Furthermore, ketone ester supplementation does not affect body composition, but shows its potential in weight management by preserving a drop in exercise capacity and REE and by improving overall stress parameters during a period of caloric restriction. These findings warrant future studies to explore the effects of intermittent exogenous ketosis in weight management in athletic populations.

## Data Availability

The raw data supporting the conclusion of this article will be made available by the authors, without undue reservation.
