# Supplementary material for: Effect of increased protein intake and exogenous ketosis on body composition, energy expenditure and exercise capacity during a hypocaloric diet in recreational female athletes
Source: Front Physiol. 2023 Jan 13;13:1063956. doi: 10.3389/fphys.2022.1063956 (PMC9880233; doi:10.3389/fphys.2022.1063956)
Supplement: Supplementary file 1 [file Table1.DOCX]

Supplementary Material

**Supplementary Table 1. Effect of increased protein intake and exogenous ketosis on handgrip strength, counter movement jump and maximal isometric knee extension torque during a hypocaloric diet.**

*Data are mean ± SEM. Subjects were involved in a 4-week hypocaloric diet (30% energy restriction, 0.8-1.0 g protein ∙ kg BW^-1^∙day^-1^) and received either placebo (PLA: n=11), an increased amount of dietary protein (PROT: n=10) or a ketone ester (KE: n=11). Handgrip strength, counter movement jump and maximal isometric knee extension torque were measured before (pretest) and at the end (posttest) of the caloric restriction period.*

|  | **PLA** | **PROT** | **KE** | P (Group) | P (Time) | P (Group x Time) |
| --- | --- | --- | --- | --- | --- | --- |
| **Handgrip strength (kg)** | | |  |  |  |  |
| Pretest | 32.6 ± 1.6 | 35.7 ± 1.6 | 35.3 ± 1.8 | 0.309 | 0.234 | 0.580 |
| Posttest | 32.8 ± 1.7 | 36.5 ± 1.3 | 35.4 ± 1.6 |  |  |  |
| **Counter movement jump (cm)** | | |  |  |  |  |
| Pretest | 26.5 ± 1.4 | 24.7 ± 1.6 | 26.0 ± 1.7 | 0.575 | 0.967 | 0.305 |
| Posttest | 26.9 ± 1.5 | 23.8 ± 2.1 | 26.4 ± 1.9 |  |  |  |
| **Maximal isometric knee extension torque (Nm)** | | | |  |  |  |
| Pretest | 156.2 ± 7.5 | 163.8 ± 10.3 | 165.0 ± 8.7 | 0.608 | 0.060 | 0.296 |
| Posttest | 155.8 ± 7.5 | 168.8 ± 10.4 | 169.9 ± 9.9 |  |  |  |
